# Immune monitoring and TCR sequencing of CD4 T cells in a long term responsive patient with metastasized pancreatic ductal carcinoma treated with individualized, neoepitope-derived multipeptide vaccines: a case report

**DOI:** 10.1186/s12967-018-1382-1

**Published:** 2018-02-06

**Authors:** Katja Sonntag, Hisayoshi Hashimoto, Matthias Eyrich, Moritz Menzel, Max Schubach, Dennis Döcker, Florian Battke, Carolina Courage, Helmut Lambertz, Rupert Handgretinger, Saskia Biskup, Karin Schilbach

**Affiliations:** 1 Department of Pediatric Hematology and Oncology, University Children’s Hospital Tübingen, Hoppe-Seyler Street 1, 72076 Tübingen, Germany; 2 Department of Pediatric Hematology, Oncology and Stem Cell Transplantation, University Medical Center Würzburg, Josef-Schneider Street 2, 97080 Würzburg, Germany; 3 Center for Genomics and Transcriptomics (CeGaT) GmbH and Practice for Human Genetics, Paul-Ehrlich-Straße 23, 72076 Tübingen, Germany; 4 Institute for Medical and Human Genetics, Charité – Universitätsmedizin Berlin, Augustenburger Platz 1, 13353 Berlin, Germany; 5 Folkhälsan Institute of Genetics, Haartmaninkatu 8, 00014 Helsinki, Finland; 6 Klinikum Garmisch-Partenkirchen GmbH, Zentrum für Innere Medizin, 82467 Garmisch-Partenkirchen, Germany; 7 University Children’s Hospital, University Medical Center Tübingen, Hoppe-Seyler-Street 1, 72076 Tübingen, Germany

**Keywords:** Pancreatic carcinoma, Therapeutic vaccines, Neoepitope-derived peptides, T-cell responses, CDR3 sequences

## Abstract

**Background:**

Cancer vaccines can effectively establish clinically relevant tumor immunity. Novel sequencing approaches rapidly identify the mutational fingerprint of tumors, thus allowing to generate personalized tumor vaccines within a few weeks from diagnosis. Here, we report the case of a 62-year-old patient receiving a four-peptide-vaccine targeting the two sole mutations of his pancreatic tumor, identified via exome sequencing.

**Methods:**

Vaccination started during chemotherapy in second complete remission and continued monthly thereafter. We tracked IFN-γ^+^ T cell responses against vaccine peptides in peripheral blood after 12, 17 and 34 vaccinations by analyzing T-cell receptor (TCR) repertoire diversity and epitope-binding regions of peptide-reactive T-cell lines and clones. By restricting analysis to sorted IFN-γ-producing T cells we could assure epitope-specificity, functionality, and T_H_1 polarization.

**Results:**

A peptide-specific T-cell response against three of the four vaccine peptides could be detected sequentially. Molecular TCR analysis revealed a broad vaccine-reactive TCR repertoire with clones of discernible specificity. Four identical or convergent TCR sequences could be identified at more than one time-point, indicating timely persistence of vaccine-reactive T cells. One dominant TCR expressing a dual TCRVα chain could be found in three T-cell clones. The observed T-cell responses possibly contributed to clinical outcome: The patient is alive 6 years after initial diagnosis and in complete remission for 4 years now.

**Conclusions:**

Therapeutic vaccination with a neoantigen-derived four-peptide vaccine resulted in a diverse and long-lasting immune response against these targets which was associated with prolonged clinical remission. These data warrant confirmation in a larger proof-of concept clinical trial.

**Electronic supplementary material:**

The online version of this article (10.1186/s12967-018-1382-1) contains supplementary material, which is available to authorized users.

## Background

Pancreatic cancer represents an aggressive cancer entity with high morbidity and mortality—especially for patients with advanced and metastatic diseases. The overall 5-year survival probability is less than 5% [1–4]. Therefore, novel therapeutic options are urgently needed. Within the past decade, enthusiasm of using vaccines as anticancer agents has revived. Data collected so far document that a variety of anticancer vaccines including cell-, DNA-, and purified component-based vaccines are capable of circumventing the poorly immunogenic and highly immunosuppressive nature of most tumors and eliciting therapeutically relevant immune responses [5, 6]. Various lines of evidence suggest that pancreatic adenocarcinoma can also induce anti-tumoral T-cell responses [7–9], thus “off-the-shelf” peptide vaccines (KRAS, Gastrin G17DT, HSP-CC-96, WT1, VEGF-R and2, hTERT, Her2/neu, KIF20A [10]), recombinant vaccines (MUC-1- and CEA-expressing poxviruses with GM-CSF), live attenuated Listeria mesothelin-expressing vaccines, irradiated whole allogenic tumor and Listeria [11], as well as inactivated whole tumor cell vaccines (Algenpantucel-L, allogeneic GM-CSF) have been evaluated for therapy in this type of cancer [12–14]. Preliminary studies yielded promising results, yet could not demonstrate significant improvement of patient survival. Nevertheless, they emphasized several critical aspects for the design of successful next-generation cancer vaccines, namely: (i) cancer vaccines should target tumor-specific antigens not expressed on healthy tissue, (ii) the applied adjuvant should potently activate antigen-presenting cells (APCs) which in turn stimulate antigen-specific cytotoxic T lymphocytes (CTLs) [15], and (iii) vaccine schedules should include strategies for breaking immunological tolerance.

Non-self-antigens like unique neo-antigens created by tumor specific mutations have hitherto been cumbersome to detect. The laborious search for tumor-specific mutations including cDNA expression cloning, serologic analysis of recombinant cDNA expression libraries (SEREX), and reverse immunological approaches [16] was dramatically simplified with the advent of next-generation sequencing (NGS) technologies. Entire cancer exomes can be sequenced and compared with healthy tissue (germline) exome, providing the fundamentally new opportunity to cover the patient’s individual aberrancy within a personalized vaccine. Such an approach integrates the tremendous heterogeneity of tumors and increases the probability of generating a tumor-specific immune response, since T cells theoretically should bind with a higher affinity to neo-antigens that have not been subject to thymic negative selection. In this context, oncogenic driver mutations are not necessarily tumor-rejecting antigens [10, 17, 18], and therapeutically useful targets may be generated from individual passenger mutations as well [19].

As the induction of cell-mediated immunity requires antigen-presentation by activated professional antigen-presenting cells (APCs) [20], vaccines must be administered in conjunction with adjuvants such as incomplete Freund’s adjuvant, diverse TLR agonists, alum, or immunostimulatory cytokines such as GM-CSF. In the present study GM-CSF was chosen, since it boosted the vaccine efficiency in the first licensed cancer vaccine Provenge [21], and improved patient outcome in phase 3 studies when applied in combination with suitable anti-tumor vaccines [22].

Considerable progress towards enhancing vaccine efficacy has been achieved by combining anti-cancer vaccines with a varied panel of therapeutics, aiming to break the immune-suppressive nature of the tumor milieu [23]. Among those agents working synergistically with immune interventions are diverse inhibitors of checkpoint molecules, targeted and/or chemo-therapies that can induce immunogenic cell death (ICD) [5, 23–25]. One of those compounds is oxaliplatin, a platinum derivative and part of the Folfirinox regimen (fluorouracil/leucovorin, irinotecan, oxaliplatin), a drug combination frequently used in the treatment of pancreatic cancer [26, 27]. This regimen combines agents that stimulate the release of danger signals, upregulates cellular tumor antigens, and induces ICD [23]. In addition, oxaliplatin reduces the expression of PD-L2, thus enhances antigen-specific proliferation and Th1 cytokine secretion [28], while 5-FU induces apoptosis selectively in MDSC and attenuates T_reg_ activity. Therefore, the Folfirinox regimen may induce synergies with immunotherapy by neutralizing immunosuppression and fueling neo-antigen-specific immunity [29–31].

Here, we characterize the T-cell response of a patient receiving a 4-peptide-vaccine targeting the two sole mutations of his tumor in an individual patient treatment approach. Our data show the ability of vaccine-peptides to elicit antigen-specific immunity against pancreatic carcinoma antigens. We demonstrate the broadness of the induced T-cell repertoire, its persistence and modulation over time, as well as novel aspects of vaccine-induced T-cell responses.

## Methods

### Patient data

**Fig. 1:**
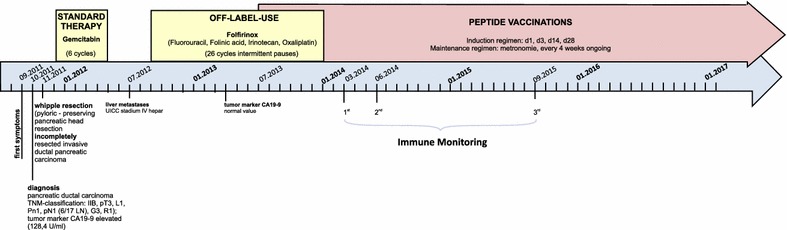
Clinical course of patient’s disease: time line of diagnoses and tumor staging, begin of treatment initiation (yellow block: polychemotherapy, red block: anti-tumor peptide vaccine) and time points of immune monitoring

A 62 year old male patient was diagnosed with pancreatic ductal carcinoma in September 2011 and subsequently underwent pylorus-preserving duodeno-cephalo-pancreatectomy in October 2011. According to UICC TNM nomenclature the tumor was classified as an incompletely resected (R1), poorly differentiated ductal adenocarcinoma Stage IIB, pT3pN1M0. According to standard treatment guidelines the patient received 6 cycles of Gemcitabine. Six months later (7/2012), a metastasis in the liver was diagnosed and probed, yet not surgically resected. Subsequently, second line chemotherapy was started 9/2012 with application of the Folfirinox regimen. 10 months later (7/2013), after 19 cycles of Folfirinox, the patient was radiologically in second complete remission, the tumor marker CA19-9 had returned to normal range and therapeutic vaccination with tumor-specific peptides was initiated. Vaccine induction regimen consisted of five peptide vaccinations combined with GM-CSF (Leukine, Berlex, Berlin, Germany) as an adjuvant on days 1, 3, 7, 14 and 28, followed by monthly vaccine boosts. No vaccine-related adverse events were observed. Folfirinox chemotherapy continued in parallel for another 7 cycles until January 2014 and was finally halted after a total of 26 cycles. Peptide vaccination is currently ongoing. The patient has been in complete remission (CR) for more than 4 years, i.e. 6 years after diagnosis. Time line of events is detailed in Fig. 1.

The patient gave his written informed consent for the collection of blood and tissue samples and additional consent was obtained for using this approach also for investigational purposes based on a biospecimen utilization protocol.

Staging, surgical resection, diagnostic procedures and adjuvant chemotherapy was applied according to standard treatment algorithms. Molecular work-up of the liver metastasis, peptide design and manufacturing was commissioned to commercial service providers by the patient. Peptide injections were carried out by the patient’s general practitioner.

### Identification of neo-antigens and peptide design

Genomic DNA was isolated from a liver metastasis biopsy of the pancreatic carcinoma and a blood sample of the patient (reference tissue). The samples were enriched for whole exome sequencing (Agilent In-Solution Technology) and sequenced on a SOLiD 5500xl next generation sequencing platform (Applied Biosystems/Life Technologies now Thermo Fisher Scientific). The sequence information derived from the tumor tissue was bioinformatically compared with the sequence information derived from the reference tissue. The called variants were then reviewed manually. One missense variant was identified, namely within the RIM1-Gene, c.[402G > A], p.[Met134Ile] (according to NM_024945). Gene expression analysis of the altered sequence on mRNA level was performed for verification (RNA isolation from fresh frozen tissue). A second mutation with ambiguous quality was found in the KIF4B-Gene. Since this mutation also represents a potential anti-tumor target, this variant was also used for neo-epitope delineation.

**Fig. 2:**
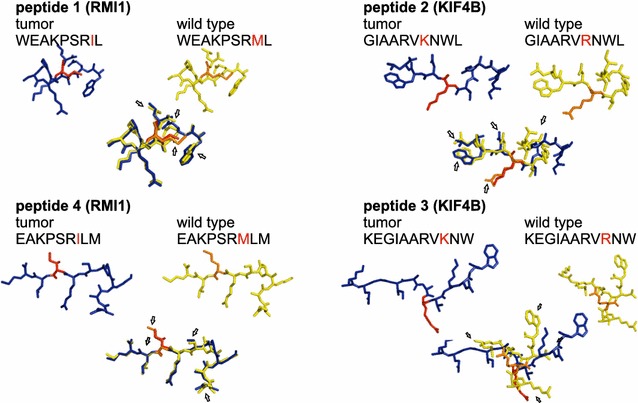
Structure of peptides covering neo- versus wild-type epitopes. Tumor-specific single nucleotide polymorphisms (orange) identified with NGS technology in the proteins RMI1 and KIF4B. Two 9-mer peptides were designed per tumor specific SNP, each peptide spanning the respective mutated sequence, yet varying at the N-terminus. Structural differences between normal (yellow) and tumor specific peptide (blue) are shown in 3D, generated with IMTEC and UCSF chimera

For the design of vaccine peptides, epitope prediction was performed with the HLA class I peptide binding algorithms NetMHC [32, 33] and SYFPEITHI [34] (Table 1). Two peptides—predicted to bind to the patients HLA class I—were designed for each neo-sequence by laying a virtual 9 amino acid frame onto the respective mutated sequence. 2 peptides differing from one another in the 5′ and 3′ end, respectively (Fig. 2) were chosen for vaccination purposes according to optimum likelihood to be presented by the patients HLA ligands (A01:01, A02:01; B18:01, B44:02; C07:01, C05:041, DRB1 11:01, DRB1 13:01 DQB1 03:01, DQB1 06:03) Peptide 1: RMI1_127–136_ epitope (WEAKPSRIL), peptide 2; KIF4B_736–747_ epitope (GIAARVKNWL), peptide 3; KIF4B_738–748_ epitope (KEGIAARVKNW), and peptide 4; RMI1_128–136_ epitope (EAKPSRILM)).

**Table 1:** The binding scores of individual peptides for the patient’s HLA class I and II haplotypes and CD1d

Peptide	Protien origin	Peptide sequence	MHC class I	Net MHC [32, 33]	Syfpeithi [34]	CD1d	Castano [55]	MHC class II	Net MHC II pan3.1 [35]
1	RIM1	WEAKPSRIL	B*18	WB	+(17)	CD1d	−	DRB1*1101	NB
			B*44	WB		CD1d	−	DRB1*1301	NB
		(WEAKPSRI)	B*44	SB	+(21)	–	−		
2	KIF4B	GIAARVKNWL	A*02	NB	+(22)	CD1d	+	DRB1*1101	WB
								DRB1*1301	WB
3	KIF4B	KEGIAARVKNW	B*44	WB	−(14)	CD1d	+	DRB1*1101	NB
								DRB1*1301	NB
		(EGIAARVKNW)	B*44	SB	−(14)	CD1d	+		
4	RIM1	EAKPSRILM	A*02	NB	−(6)	CD1d	−	DRB1*1101	NB
								DRB1*1301	NB

The binding scores of individual peptides for the patient’s HLA haplotypes were determined via NetMHC [32, 33], SYFPEITHI [34], CD1d-binding algorithm according to “Castano” (1-4-7 rule) [55] and NetMHCIIpan version 3.1 [35]

Then likelihood for presentation is given as “+” and “−” respectively; SYFPEITHI half max scores regarding MHC class I presentation are given in brackets. Mutations in the peptides are indicated by underline

*WB* weak binder, *SB* strong binder, *NB* no binding predicted

### Application of the vaccine

Vaccination regimen started with a priming phase by vaccinating the patient on days 1, 3, 8, 15 and 29, and then continued by monthly boost vaccines for maintenance. The peptide cocktail was applied intracutaneously, the adjuvant GM-CSF (sargramostin 250 µg/injection, Leukine^®^) was applied subcutaneously.

### Vaccination peptide synthesis and vaccine formulation

Vaccination peptides were ordered commercially (American Peptide Company) by the patient. Before injection, the lyophilized peptides were dissolved in DMSO (Hybri-Max, sterile filtered, Sigma-Aldrich) and further diluted in water (Aqua ad injectabilia, B. Braun, Melsungen, Germany) to a final concentration of 0.8 mg/ml per peptide (400 µg/500 µl vaccination dose per peptide).

### Immune monitoring

#### Enrichment of peptide-specific T cells

Immune monitoring was carried out at three defined time points: *IM1* 28 weeks, *IM2* 44 weeks, and *IM3* 108 weeks after initiation of peptide vaccines (after having received 8, 12 and 28 vaccinations). At the respective time points, patient PBMCs from freshly drawn heparinized blood were isolated with Ficoll Isopaque density gradient centrifugation (Pharmacia, Uppsala, Sweden) and cultured in RPMI1640 (10% FBS, P/S, Glu) supplemented with low dose IL-2 (20U/ml), IL-7 (2 ng/ml) and vaccine peptide- (10 µg/ml) or PBS-pulsed (controls) autologous feeder cells to bias survival of potentially in vivo primed T cells. Fresh medium containing 10% FBS supplemented with IL-2 (20U/ml) and IL-7 (2 ng/ml) was provided twice per week, whereas peptide stimulus was given only at day 7 of culture. At day 12, cells were restimulated with peptide-pulsed (10 µg/ml) PBMCs and T cells responding to vaccine-peptide stimulus with IFN-γ production were isolated 5 h later using IFN-γ Secretion Assay Kit (Miltenyi Biotech, Germany) according to the manufacturer’s instructions. Isolated cells were analyzed for the complexity of their T-cell receptor repertoire and epitope-binding region (CDR3 sequence). In *IM*1, PBMCs were stimulated with a pool of all four peptides, in *IM*2 the four vaccine peptides were analyzed separately, in *IM*3 cloning experiments were performed which allowed a more detailed identification of peptide-specific TCRs. CDR3 size spectratyping of unmanipulated peripheral T cells and direct sequencing of single prominent peaks was performed at all time points. Additional file 1: Fig. S1 provides an overview of immune monitoring techniques.

#### Cloning of neo-antigen-specific T cells for identification of TCR heterodimers

IFN-γ -selected T cells were expanded for 1 week in standard RPMI1640 supplemented with IL-7 (10 ng/ml), IL-2 (100 U/ml), and IL-15 (10 ng/ml) and subsequently cloned following standard limiting dilution procedure (0.3 cells in a 96-round-bottom-well with 10^5^ feeder cells (80 Gy-irradiated autologous PBMCs). After 3 weeks of cloning culture—allogeneic PBMCs from a 9/10 HLA-matched healthy donor (A02:01, A02:01; B18:01, B44:02; C07:01, C05:041; DRB1 11:01, DRB1 13:01; DQB1 03:01, DQB1 06:03) were used as peptide-presenting feeder cells. T-cell cloning culture was supplemented with fresh medium, IL-7 (10 ng/ml), IL-2 (50U/ml), and IL-15 (10 ng/ml) twice per week and peptide-pulsed feeder cells once per week.

Peptide specificity of established clones was evaluated using peptide-pulsed monocytes from a HLA-matched healthy donor. Presenting monocytes had been highly enriched by MACS with αCD14 microbeads (Miltenyi Biotec; Bergisch-Gladbach,Germany) to prevent donor T-cell contamination. IFN-γ production of T-cells clones in response to peptide-presenting monocytes was quantified by routine intracellular FACS staining procedures.

### Molecular methods

#### RNA extraction, cDNA synthesis

RNA was extracted with the RNeasy Mini Kit (Qiagen, Hilden, Germany) and reverse transcribed using the Superscript III First Strand Synthesis Super Mix (Life Technology, Germany) as recommended by the manufacturer.

#### TCR Vα- and Vβ-repertoire spectratyping

TCR repertoire complexity was analyzed by CDR3 size spectratyping in 32 (TCRα) and 24 (TCRβ) PCR reactions as published previously [36]. Amplified PCR products were size-fractionated by capillary gel electrophoresis using the ABI model 3130 Genetic Analyzer, and data analyzed with GeneMapper v4.0 (Applied Biosystems).

#### Identification of the epitope-binding region of TCRs from neo-antigen-specific T cells

Single peaks in TCRVα- and Vβ-repertoire spectratype analysis were subjected to direct sequencing approaches for determination of CDR3 amino acid sequences using the BigDye^®^ Terminator v3.1 Cycle Sequencing Kit (Life Technologies, Germany). Sequences were read in an ABI3130 Genetic Analyzer and matched with IMGT, NCBI Blast and Emboss databases.

Furthermore, selected clones were analyzed by flow cytometry for TCRVβ expression using the IOTest^®^ Beta Mark Kit (Beckman Coulter, Germany).

#### Real time PCR

For (quantitative) analysis of cytokine and transcription factor expression, perforin-, granzyme B-, class I-restricted T cell-associated molecule (CRTAM)- and GAPDH-specific primers were used in combination with the SYBR Green kit (Promega, USA) in a BioRad C1000 Thermal cycler/CFX96 real-time System (BioRad, Germany). GAPDH was used as reference gene. Briefly, cDNA was added to a final volume of 10 μl/reaction containing 1 × SYBR Green PCR Master Mix (Promega, USA) and 100 nM of each primer. Thermal cycling conditions were: denaturation at 95 °C 10 min, 40 cycles: 95 °C/30′′, 60 °C/30′′ and 72 °C, 1 min for elongation. Primer sequences were: CRTAM-for: 5′-CCTCTCAAGACCCACAGCAG-3′, CRTAM-rev: 5′-AATGAGGAA-GGACACCAGCG-3′, perforin-for, 5′-ACCAGCAATGTGCATGTGTCTG-3′ and perforin–rev: 5′-GGCCCTCTTGAAGTCAGGGT-3′ [37], GrzB for: 5′-TTCGTGCTGA-CAGCTGCTCACT-3′ and GrzB-rev, 5′-CTCTCCAGCTGCAGTAGCA-TGA-3′ [38], GAPDH-for: 5′-CCACATCGCTCAGACACCAT-3′ and GAPDH-rev: 5′-GGCAACAA-TATCCACTTTACCAGACT-3′ (RTPrimerDB ID 2053).

#### Flow cytometry

Cells were stained according to standard procedures using the following antibodies (clone, manufacturer): PerCP/APC-CD3 (SK7, BD), Vioblue-CD4 (VIT4, Miltenyi Biotec Germany), APC-H7-CD8α (SK1, BD), FITC/APC-CD62L (LT-TD180, ImmunoTools), APC-CD25 (2A3, BD Pharmingen), PE-CF594-A-CCR7 (150503, BD), CD45RO, PE-Cy7-CD45RA (H/100, BD), CD27, UV1-A-CD25, APC-CD69 (FN50, Biolegend), CD276, UV3-CD28, CD95. Intracellular fixation/permeabilization kit (eBioscience) and Brilliant-Violet 785-TNF-α (MAb11), PE-IFN-γ (B27) were used for intracellular cytokine staining according to the manufacturer’s instructions. Dead cells were excluded via Alexa Fluor 350 (Invitrogen) or via Zombie Aqua™ (Biolegend). PBMCs were pretreated with FcR Blocking Reagent (Miltenyi Biotec) according to the manufacturer’s recommendations. Samples were analyzed on a LSR II or FACS Canto II with FACS Diva software (BD Biosciences).

#### Statistical analysis

Data were analyzed with Prism 6.0 (GraphPad Software, Inc) and Student’s t test. P < 0.05 was considered statistically significant.

## Results

### Patient data, vaccine application and clinical outcome

After receiving state-of-the-art oncological treatment for primary and relapsed pancreatic carcinoma, immunotherapeutic interventions were initiated in July 2013 when routine staging (sonography, CT-scan, MRI) did not show any residual tumor, neither at the primary nor at the metastatic site (second complete remission). The tumor was found to be positive for CA19-9 and blood levels correlated with the disease course. CA19-9 levels have remained within normal ranges since March 2013. Chemotherapy was terminated 27 months after disease onset (Fig. 1). Currently, 64 months after initial diagnosis and 43 months after initialization of vaccine therapy, the patient is in good health and fully active (ECOG 0). The latest routine examination (physical examination and laboratory test in March 2017, CT in Jun 2016 and sonography in November 2016) showed no evidence of disease.

### Overall TCR repertoire complexity increases during vaccination

TCRVα and Vβ-chain repertoires of the unmanipulated peripheral T-cell pool at *IM1* showed a reduced complexity most likely as a consequence of long-term chemotherapy. Numerous families in both the TCRVα and Vβ chain repertoires, exhibited profound skewing indicating limited diversity and clonal expansions. Accordingly, TCRVα 8-1, 14DV4, 21, 30, and TCRVβ 6-5, 12-3, 21-1 sequences—obtained by direct sequencing of singular peaks (Additional file 2: Fig. S2)—showed CDR3 motifs similar to published TCRs specific for CMV (TCRVα 8-1, 30, TCRVβ 12-3, 21-1) (Table 2) and mycolic acid (TCRVβ 6-5) (a cell wall component of several bacteria including mycobacterium tuberculosis) and thus can be regarded as public TCR motifs [39–41].

**Table 2:** CDR3 sequences before short term culture and CMV specific CDR3 sequences

CDR3 sequences obtained by direct sequencing of dominant, singular peaks in CDR3 size spectratyping of peripheral T cells that seemed to dominate the TCR repertoire without prior stimulation of expansion. Public, CMV-specific CDR3 sequences published previously by Zvyagin et al. are marked in grey [39]

**Fig. 3:**
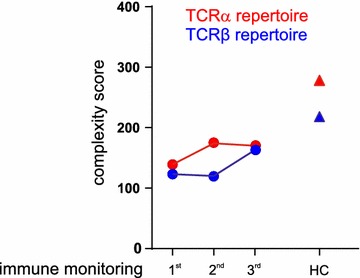
Complexity score of peripheral blood TCRVα and TCR-Vβ repertoire in our patient at the 1st, 2nd and 3rd immune monitoring as well as in healthy controls (HC). The complexity of Vβ- and Vα-chain repertoires was determined by counting the number of peaks in spectratype analysis. A score of 8 describes a normal CDR3 size variability of 8–10 peaks per Gaussian curve, a score of 1 refers to profiles showing single peak, 0 describes the absence of peaks. The overall TCR complexity (complexity score) is the sum of 26 individual TCR Vβ- or 34 TCRVα family scores respectively (with a maximum of 26 × 8 = 208 for the β-, and 34 × 8 = 272 for the Vα families)

Since the patient had no documented infection with CMV or mycobacteriaceae at that time, detection of these public TCRs rather reflects past infectious episodes with these microorganisms, which become more prominent with increasing age and under lymphodepleting chemotherapy [42]. The overall complexity of the TCRVα and Vβ chain repertoires slightly increased over time after cessation of Folfirinox (Fig. 3).

### Detection of vaccine-reactive T cells at *IM1*

**Fig. 4:**
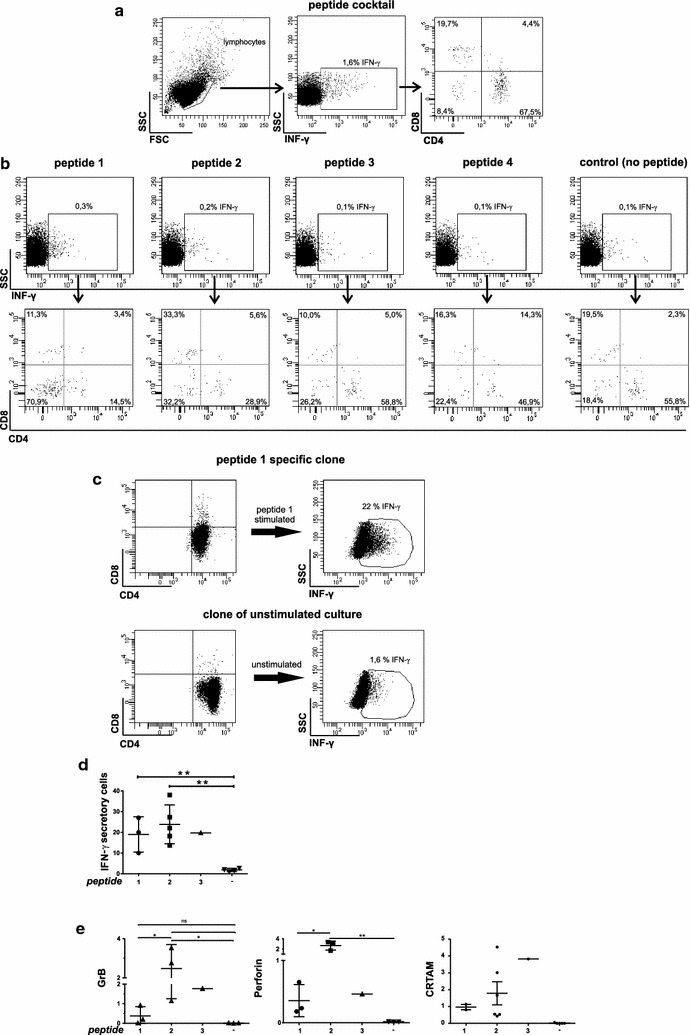
Ex vivo peptide stimulation assays of T cells. T cells were pulsed with peptide, expanded and restimulated in short-term in vitro culture. Cells were gated on live-cells, lymphocytic fraction, CD3^+^ cells. **a** At IM1, IFN-γ secretion of T cells was measured after stimulation with a peptide cocktail containing all four peptides 4. **b** At IM2, T cells were stimulated with single peptides, including a no-peptide control, respectively. **c** IFN-γ-secretion and phenotype of a T-cell clone (#33) responsive to peptide 1 obtained from peripheral T cells at IM3. **d** Percentage of cells from clonal populations of defined specificity responding to their peptide with IFN-γ^+^ production. **e** mRNA expression of granzymeB, perforin and CRTAM in peptide-specific clones from IM3 measured by RT-PCR. Expression of the target gene was normalized to expression of human GAPDH. *P < 0.05, **P < 0.01

To test whether the priming phase of the vaccine has resulted in a detectable vaccine-reactive T-cell response, PBMCs drawn 28 weeks after initiation of vaccination (i.e. after 8 vaccines) were cultivated ex vivo in the presence of vaccine peptides and low dose IL-2, in the absence of a mitogen. Such limiting growth conditions only activate and expand memory T cells since memory T cells have an increased sensitivity for TCR stimulation, are more sensitive to low doses of antigen and have an increased proliferative potential even without excessive support of cytokines [43]. Moreover, the number of mature dendritic cells that is necessary to efficiently stimulate and prime naïve T cells in context with costimulatory molecules is low in PBMCs thus naïve T cells get stimulated simply with their cognate antigen alone, which makes them refractory to further stimulus, hyporesponsive and anergic [44, 45]. Thus the short term culture protocol impedes in vitro priming of naïve T cells, which could lead to false positive results. After expansion and restimulation, a total of 1.6% of T cells were responsive to the peptide pool with IFN–γ^+^ production, indicating a peptide-specific response (Fig. 4a). Interestingly, the majority of responding T cells were CD4^+^ (67.5%), the remaining CD8^+^ (19.7%) or double negative (Fig. 4a).

### Vaccine peptides contribute differently to the T-cell response, IM2

Immune monitoring at time point IM2 (44 weeks after initial vaccination) was designed to discriminate how the 4 vaccine peptides contributed to the induced T-cell response. This time, PBMCs were stimulated with every single peptide in short term pre-cultures separately. Peptide 1 boosted the biggest share of IFN-γ^+^ T cells (0.3% IFN-γ^+^ cells/CD3^+^), followed by peptide 2 (0.2% IFN-γ^+^ cells/CD3^+^), whereas peptide 3 and 4 showed responses only around the background level (each 0.1% IFN-γ^+^ cells/CD3^+^) (Fig. 4b). This demonstrates that vaccine peptides were able to induce specific T-cell immunity yet quantitatively differed in their ability. Of note, peptides 1 and 2 had higher SYFPEITHI scores for HLA A*02:01 and B*44:02 than peptides 3 and 4 (Table 1). Only peptide 2 was also predicted as a binder for HLA DRB1*1101 and HLA DRB1*1301 (Table 1). Again, IFN-γ^+^ T cells were largely CD4^+^ dominated except for peptide 1 responding T cells, which—quite unexpectedly—were mostly (70%) CD4-/CD8-double negative (DN) T cells. γδ T cells–T cells that belong to innate immunity—lack a coreceptor and without the restriction for HLA presentation can directly respond to epitopes in proteins in either three dimensional as well as linear unfolded form, and also to soluble proteins or small peptide antigens [46]. Intriguingly, the Tetanus toxin-derived peptide *Clostridium tetani*_1235–1246_ (GNAFNNLDRILR), for which γδ TCR-dependent responses have been reported [47], shares a 3 amino acid motif with the vaccine peptide WEAKSPRIL. This motif length is sufficient in size to provoke a TCR mediated γδ T cell peptide response [48]. Therefore it is legitimate to assume that the DN T cells responding to peptides are γδ T cells.

### Molecular characterization of peptide-specific T cells, *IM1*-*3*

In order to molecularly characterize the T-cell response against the vaccine on the CDR3 sequence level, we purified IFN-γ-responsive T-cells from *IM1* and *IM2* after expansion and restimulation in short-term culture via an IFN-γ-capture assay. These putatively peptide-specific T cells were then subject to CDR3 size spectratyping, direct sequencing of prominent peaks and bioinformatic data analysis. Since the genetic material obtained in this way was very limited and precluded more *in*-*depth* analyzes, at *IM3* we generated T-cell clones against the 4 peptides using pre-clonal stimulation, IFN-γ-capture, limiting dilution and subsequent clonal expansion. Peptide 1 fostered 19 clones, peptide 2 8 clones, peptide 3 1 clone, no clone could be obtained with peptide 4. Vaccine-peptide specific clones were cultivated for another 3 weeks to obtain sufficient numbers for further analysis. Finally, 10 clones could be harvested in sufficient numbers for further analysis (Table 3 lower panel).

**Table 3:** TCRVα and -Vβ CDR3 sequences of peptide-specific T cells at IM1, IM2 and IM3

Vertical brackets mark identical (“i”) or convergent CDR3 formation (“c”), the latter resulting from combination of different variable and joining regions but identical N nucleotides coding for the same amino acids

*Seq* sequence; yellow germline encoded, pink dual TCR(2 Vα chains, 1 Vβ chain), red information of clone IDs but NOT sequence IDs

*Amino acids in the “N” region position in these CDR3 sequences are germline encoded. CDR3 sequences from IFN-γ^neg^ cell fraction of IM1 were identical with CDR3 sequences identified before immune monitoring 1, 2 and 3 (Table 2). CMV specific sequences are marked in grey

T-cell clones were exclusively CD4^+^ (Fig. 4c), enriched for IFN-γ responsiveness upon restimulation with the cognate peptide (Fig. 4c, d) and yielded unequivocally singular peaks in CDR3 size spectratypes (not shown). Phenotypically, T-cell clones expressed high levels of activation markers such as CD25, CD69, and HLA-DR (Additional file 3: Fig. S3), and higher mRNA levels of granzymeB, perforin, and CRTAM—that determines the CD4+ cytotoxic T lymphocyte lineage [49]—than clones established in control cultures without peptide (Fig. 4e). Interestingly, the vast majority of T-cell clones in control cultures carried the identical public TCR Vα39 Vβ6-2, known to be specific for mycolic acid [41], (Additional file 4: Fig. S4), suggesting a high precursor frequency in peripheral blood and a significant survival advantage for these T-cells under the given culture conditions. Vaccine-peptide specific T cells were negative for Foxp3 and displayed a terminally differentiated effector T cell phenotype (T_EMRA_, CCR7^−^, CD45RA^+^) (Additional file 5: Figure S5a) [50]. In contrast, clones obtained from control cultures without peptide exhibited an effector memory phenotype (CD45RO^+^, CD45RA^−^, CD62L^−^, CCR7^−^, CD27^−^) (Additional file 5: Figure S5b).

The epitope-binding CDR3 regions of the identified TCRs were of rather short length (median of 3 N-nucleotides, Table 3) and positively charged with the exception of TCRs reactive to peptide 1. We were able to identify two TCR sequences, with *identical* TCRVα- and TCRVβ-derived CDR3 sequences at two time points indicating persistence of vaccine-specific T-cell responses: TCRVβ18 bearing seqID 68 (*IM*2) and clone 17 (*IM*3), as well as TCRVβ14 bearing seqID 69 (*IM*2) and clone 42 (*IM*3). TCRs that share a common intermediate segment of the CDR3 sequence yet are flanked by different V- and J-gene segments are termed convergent CDR3 formations. Two *convergent* CDR3 formations could be detected at least at two sequential time points: seqID 31 (*IM*1) and seqID 46 (*IM*2), as well as seqID 44 *(IM*2) and clone 42 *(IM*3), again indicating clonal persistence. Whereas the two convergent TCRVα sequences were most likely stimulated by peptide 1, the two identical CDR3 sequences unexpectedly were stimulated by peptide 3 in *IM*2 and by peptide 1 and 2 in *IM*3. Peptides 1 and 3 are clearly different, yet are both predicted to bind to HLA-B44 and show identical amino acids in anchor positions 2 (E) and 7 (R) [34]. Hydrophobic peptides 2 and 3 both bind to CD1d. Peptides 1 and 2 with the highest SYFPEITHI scores and NetMHCIIpan predictions induced most of the detectable CDR3 sequences and the highest number of clones (Table 1).

Three germline encoded CDR3 sequences (i.e. recombination of VD(J) gene segments without addition of N-nucleotides) were identified in peptide 2 (Seq. ID 46, 50) and peptide 1 (Seq. ID 44) (Table 3) pulsed cultures.

Three of four clones (35, 36, and 54) specific for peptide 1 expressed two TCRVα chains combined with one TCRVβ chain, respectively (Table 3), whereas peptide 2 stimulated five clones with singular TCRVα and -Vβ chains.

## Discussion

Identification of peptide-reactive T cells within the huge repertoire of existing TCR sequences of a human individual remains a major challenge. NGS approaches have significantly improved the sensitivity of clonality assays, e.g. in monitoring of minimal residual disease of lymphoid malignancies [51] or in tracking of known sequences of hypervariable regions. High-throughput sequencing may allow a better estimation of the actual frequency and redundancy of T-cell clonotypes, however, it is not suitable for deciphering vaccine-induced T-cell responses. Monitoring of immune responses after vaccination requires the correlation of identified TCR sequences with their specificity [52]. Moreover, more in depth analyses of peptide-reactive T cells such as the determination of TCRVα- and Vβ-chain pairing, potential dual TCR expression, phenotypic characterization of markers such as Foxp3 or CRTAM which distinguish different functional states of T cells is not possible by using NGS approaches alone. In order to circumvent these limitations of large scale, non-supervised TCR sequencing, we focused on tracking defined T-cell responses against neo-epitope peptides after short pre-culture and expansion of patient PBMCs in the presence of vaccine peptides, a standard procedure used for the detection of epitope specific T cells [53]. Only T cells responding to the peptide stimulus with IFN-γ release were isolated and used for further examination. By relying on IFN-γ production in response to a stimulus we could ascertain the specificity of these T cells, their functionality and T_H_1 phenotype. The separation of the peptide-reactive from non-specific background T cells allowed the enumeration of unique TCRVα and -Vβ sequences, an estimation on the diversity of induced TCR sequences, and tracking of clonal persistence over time. The technique of IFN-γ -capture assays is widely used for isolation and transfer of antigen-specific immunity, and T-cells sorted according to their IFN-γ secretion have demonstrated to be highly specific and functional, even in the clinical setting [54].

Our results demonstrated a discernible TCR-repertoire responsive to the vaccine peptides. Besides a consistency of the IFN-γ^+^ TCR repertoire in terms of net charge we detected two identical and two convergent CDR3 sequences at more than 1 time point, prodding persistent, vaccine-specific immunity. Furthermore, formation of convergent sequences against a common target could indicate strong immunogenicity of these neoepitope-derived peptides and indeed, three of these repetitive sequences could be attributed to the same peptide motif. However, one identical sequence was found in T cells reactive for peptide 1 (RMI1 mutation) and peptide 3 (KIF4B mutation). Whether sharing of the same presenting MHC molecule (HLA-B*44) or identical amino acids at anchor positions 2 and 7 contributed to this observation remains unclear. In general, broadness and diversity of peptide-reactive repertoires seemed to correspond with the MHC class I binding affinity based on SYFPEITHI scores [34]: strong binder peptides 1 and 2 yielded more CDR3 sequences than weak binder peptides 3 and 4. Whether peptide presentation via CD1d (binds hydrophobic peptides with hydrophobic amino acids at anchor positions 1-4-7) [55] played a role for peptides 2 and 3 presentation was not assessed in our assays. In addition, only peptide 2 was also predicted as a weak binder for both of the patient’s HLA DRB MHC class II molecules (Table 1). This likelihood for being presented by HLA class I and II and CD1d goes well with the broadest TCR repertoire and a persistent CDR3 sequence motif induced by peptide 2.

The majority of identified T cells expressed single TCRVα- and -Vβ chains. Moreover, at *IM*3 we could identify a neo-antigen specific dual TCR, consisting of two alpha chains paired with one beta chain, in three clones. A dual TCR is able to bind two specificities, of which one possibly has not undergone thymic selection [56]. Dual TCRs account for up to 30% of the normal T-cell repertoire in humans [57, 58] and in mice [59–62], and are regarded as rather beneficial for the host since they may help to generate more vigorous responses to problematic antigens [63]. Dual TCRs can endow T cells with an intrinsic property for alloreactivity but do not necessarily confer increased susceptibility for autoimmunity [64]. Interestingly, dual TCRs have been described as one of two possible mechanisms by which CD4^+^ T cells expressing a nominally MHC class I-restricted TCR can develop [65]. As a matter of fact, although class I restricted peptides were used in this study, the vaccine-responsive T cells were mainly CD4^+^ and expressed CRTAM whose intracellular signaling is required for the induction of CD4^+^ CTLs [49]. CRTAM^+^ CD4^+^ T cells have been described to possess characteristics of both CD4^+^ and CD8^+^ T cells, secrete IFN-γ, exhibit a CTL-related gene signature, such as eomesodermin (Eomes), granzymeB, and perforin, and traffic to sites of inflammation [49]. Thus, our data indicate that CD4^+^ CTLs may play a role in vaccine-induced TCR repertoires, a finding that warrants further investigation in other vaccine settings as well as in preclinical models.

From a clinical perspective, the patient continues to show a remarkable long complete remission after standard therapy for a metastasized pancreatic ductal carcinoma. Since the patient was already in second complete remission when the neoepitope-derived vaccine was initiated, we cannot draw definite conclusions whether and to what extent the induced tumor-specific immune response contributed to long-term survival. Of note, induction of the immune response and the ongoing remission was achieved without adverse events from the vaccine, a frequent finding with therapeutic vaccines. In contrast, checkpoint inhibitors like CTLA-4 or PD-L1, which may boost but cannot induce anti-tumor immune responses, are frequently associated with significant adverse events, which can be dose limiting [66]. The important question, whether checkpoint blockade could enhance vaccine-induced immune responses has to be addressed in further clinical trials.

## Conclusions

We report a remarkable long-term remission in a patient with advanced pancreatic cancer, who received an individualized four peptide vaccine based on in silico predicted peptide motifs from the two sole mutations in his cancer, i.e. in the proteins RIM1 and KIF4B. Moderate to strong cytoplasmic and/or nuclear expression of these proteins has been documented in pancreatic cancer before [67]. Three of the four peptides elicited detectable IFN-γ^+^ CTL responses which were CD4^+^ dominated. On the molecular level, both transient and persistent peptide-specific TCR CDR3 sequences could be identified. Our data show that an individualized, neoepitope-derived therapeutic vaccine *is* able to induce a tumor-specific immune response with a traceable molecular signature. This detailed immune monitoring program covering functional and molecular aspects of T-cell biology provides deepened insight into the mechanisms driving cancer vaccine responses and emphasizes the potential of personalized vaccines as a promising therapeutic strategy in maintaining long-term remissions, which have to be tested in controlled clinical trials.

## Additional files

**Additional file 1: Figure S1.** Work flow of immune monitoring techniques.

**Additional file 2: Figure S2.** Activation markers expressed by vaccine-reactive T cell clones at IM3. T cells responding to vaccine peptide stimulation with IFN-γ^+^ production were analyzed for their expression of the activation markers CD25, CD69 and HLA-DR by flow cytometry.

**Additional file 3: Figure S3.** Spectratype analysis of patient PBMCs without prior short term culture. TCR repertoire analysis including 34 TCRα and 24 TCRβ families. Numbers indicate CDR3 sequencing data gained in direct sequencing approaches (see Table 3). Peak numbers marked in grey harbor CMV-specific sequences.

**Additional file 4: Figure S4.** Clonality analysis of 21 control cultures (without peptide stimulation) at *IM*3 was performed by flow cytometry. All control clones were found to express all the same TCRVβ chain (BV 6-2), confirming molecular data, indicating a common, high-frequency progenitor in peripheral blood. (a) shows representative flow cytometry data of clone #6. Individual TCRβ antibody staining are achieved by combining 3 TCR Vβ-specific reagents in a single test using only two colors for mab conjugation: one TCR Vβ antibody is conjugated to FITC, another one to PE, and the third to both FITC and PE. In this way, the third Vβ-stained population shows up in the diagonal of the upper right quadrant in a FL1/FL2 histogram. The CDR3 sequence of the analyzed clone (b) is identical to a TCR sequence specific for mycolic acid [41].

**Additional file 5: Figure S5.** Peptide-responsive clones at *IM*3 all displayed an CD45RA^+^ effector memory T_EMRA_ phenotype. One representative example (clone #54) is shown in (a). In contrast, clones obtained from control cultures without peptide stimulation expressed a CD45RA^neg^ effector memory phenotype, data by clone #6 are shown in (b).
