# Supplementary figures and images for: Immune monitoring and TCR sequencing of CD4 T cells in a long term responsive patient with metastasized pancreatic ductal carcinoma treated with individualized, neoepitope-derived multipeptide vaccines: a case report
Source: J Transl Med. 2018 Feb 6;16:23. doi: 10.1186/s12967-018-1382-1 (PMC5801813; doi:10.1186/s12967-018-1382-1)

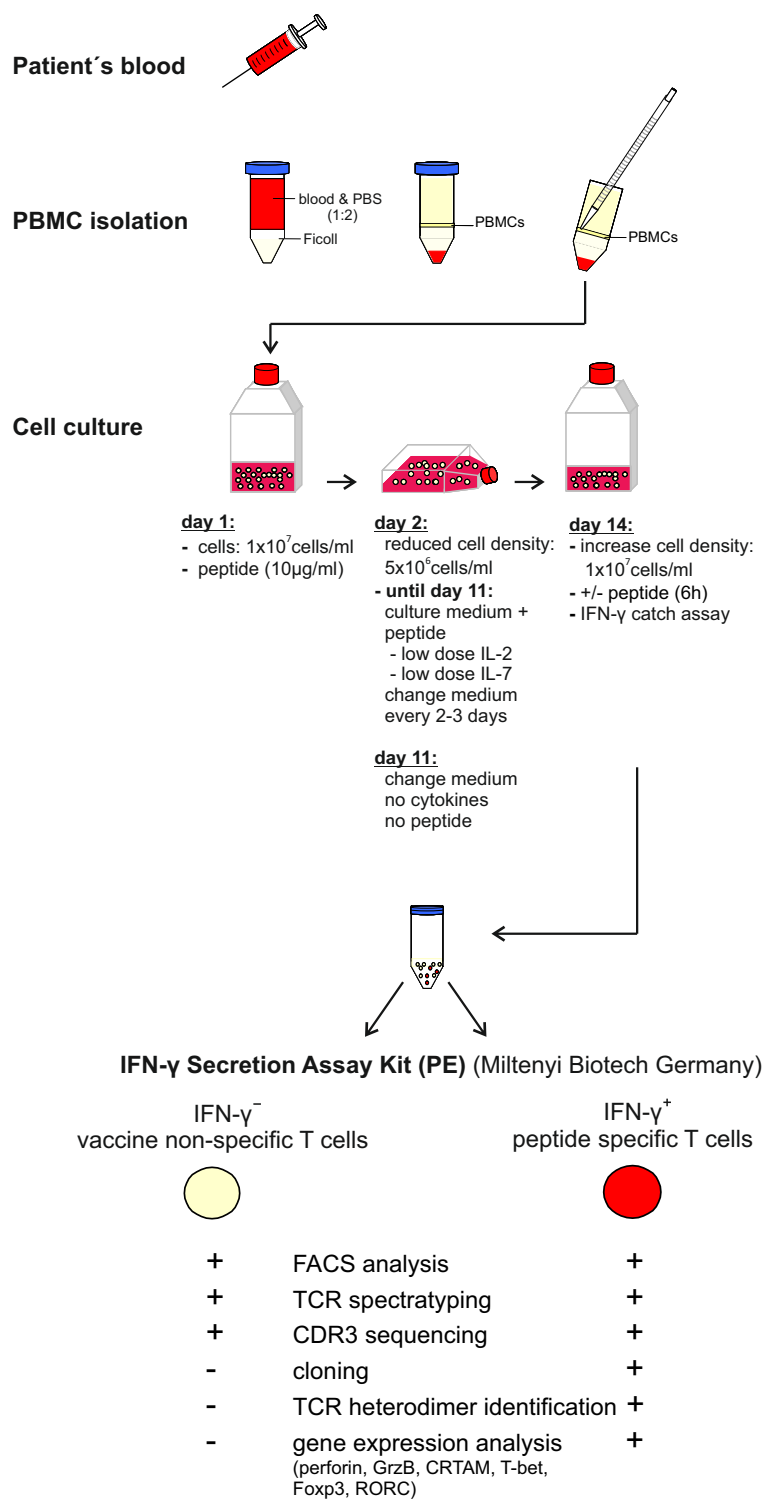

Supplement: Supplementary file 1 — Additional file 1: Figure S1. Work flow of immune monitoring techniques. [file 12967_2018_1382_MOESM1_ESM.pdf]

# TCR variable segments of locus (before immune monitoring)

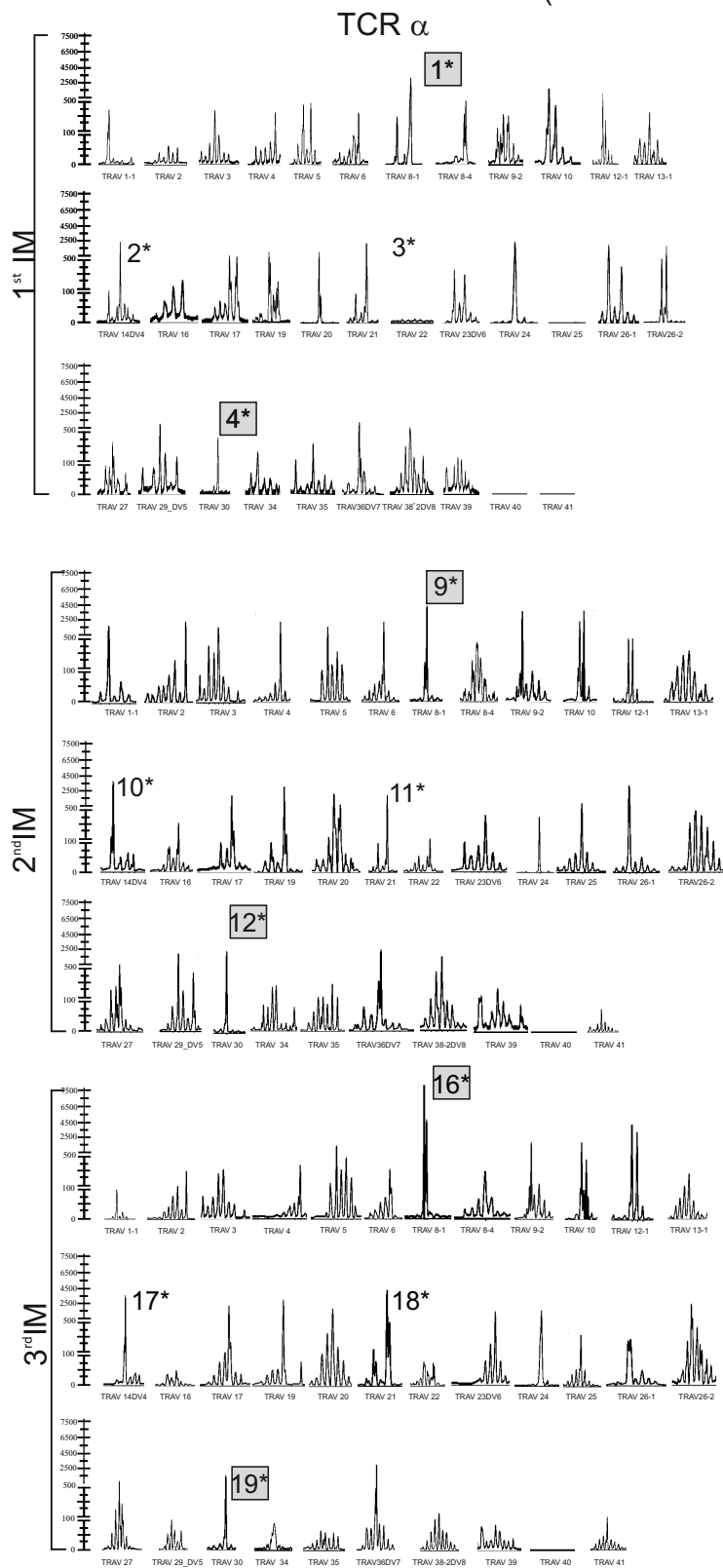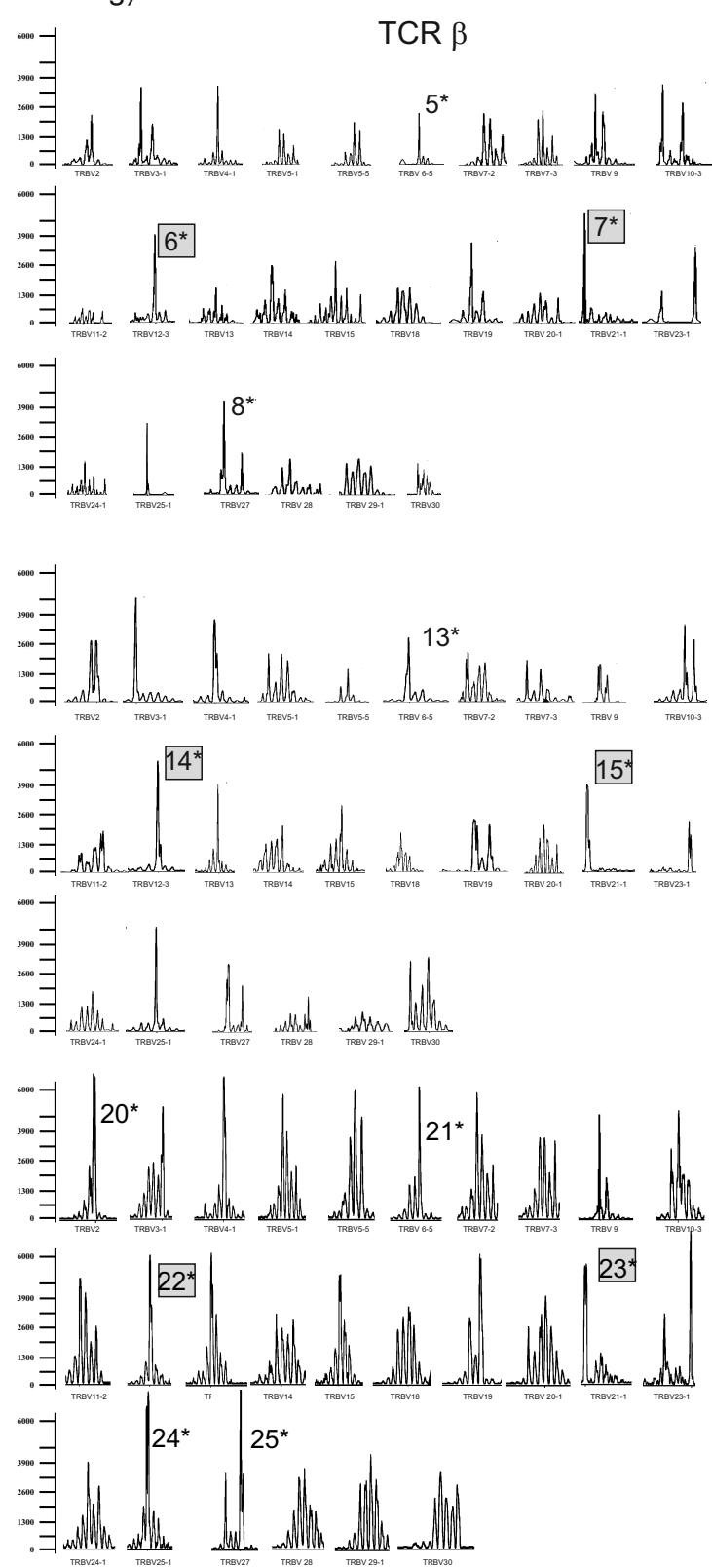

Supplement: Supplementary file 2 — Additional file 2: Figure S2. Activation markers expressed by vaccine-reactive T cell clones at IM3. T cells responding to vaccine peptide stimulation with IFN-γ+ production were analyzed for their expression of the activation markers CD25, CD69 and HLA-DR by flow cytometry. [file 12967_2018_1382_MOESM2_ESM.pdf]

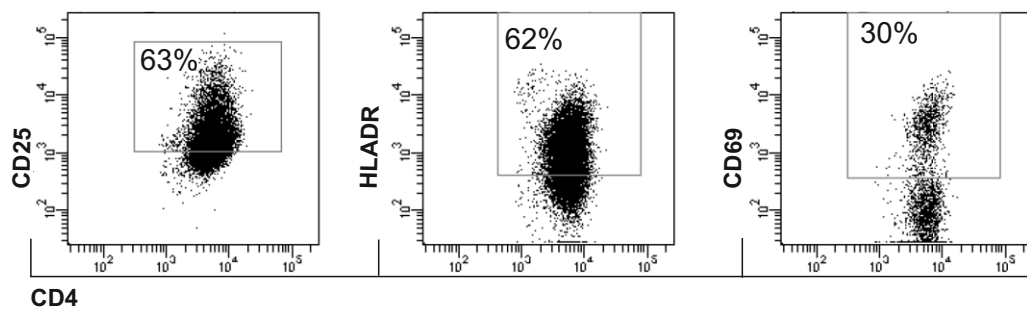

Supplement: Supplementary file 3 — Additional file 3: Figure S3. Spectratype analysis of patient PBMCs without prior short term culture. TCR repertoire analysis including 34 TCRα and 24 TCRβ families. Numbers indicate CDR3 sequencing data gained in direct sequencing approaches (see Table 3). Peak numbers marked in grey harbor CMV-specific sequences. [file 12967_2018_1382_MOESM3_ESM.pdf]
