# Supplementary material for: Immune monitoring and TCR sequencing of CD4 T cells in a long term responsive patient with metastasized pancreatic ductal carcinoma treated with individualized, neoepitope-derived multipeptide vaccines: a case report
Source: J Transl Med. 2018 Feb 6;16:23. doi: 10.1186/s12967-018-1382-1 (PMC5801813; doi:10.1186/s12967-018-1382-1)
Supplement: Supplementary file 4 — Additional file 4: Figure S4. Clonality analysis of 21 control cultures (without peptide stimulation) at IM3 was performed by flow cytometry. All control clones were found to express all the same TCRVβ chain (BV 6-2), confirming molecular data, indicating a common, high-frequency progenitor in peripheral blood. (a) shows representative flow cytometry data of clone #6. Individual TCRβ antibody staining are achieved by combining 3 TCR Vβ-specific reagents in a single test using only two colors for mab conjugation: one TCR Vβ antibody is conjugated to FITC, another one to PE, and the third to both FITC and PE. In this way, the third Vβ-stained population shows up in the diagonal of the upper right quadrant in a FL1/FL2 histogram. The CDR3 sequence of the analyzed clone (b) is identical to a TCR sequence specific for mycolic acid [41]. [file 12967_2018_1382_MOESM4_ESM.pdf]

a

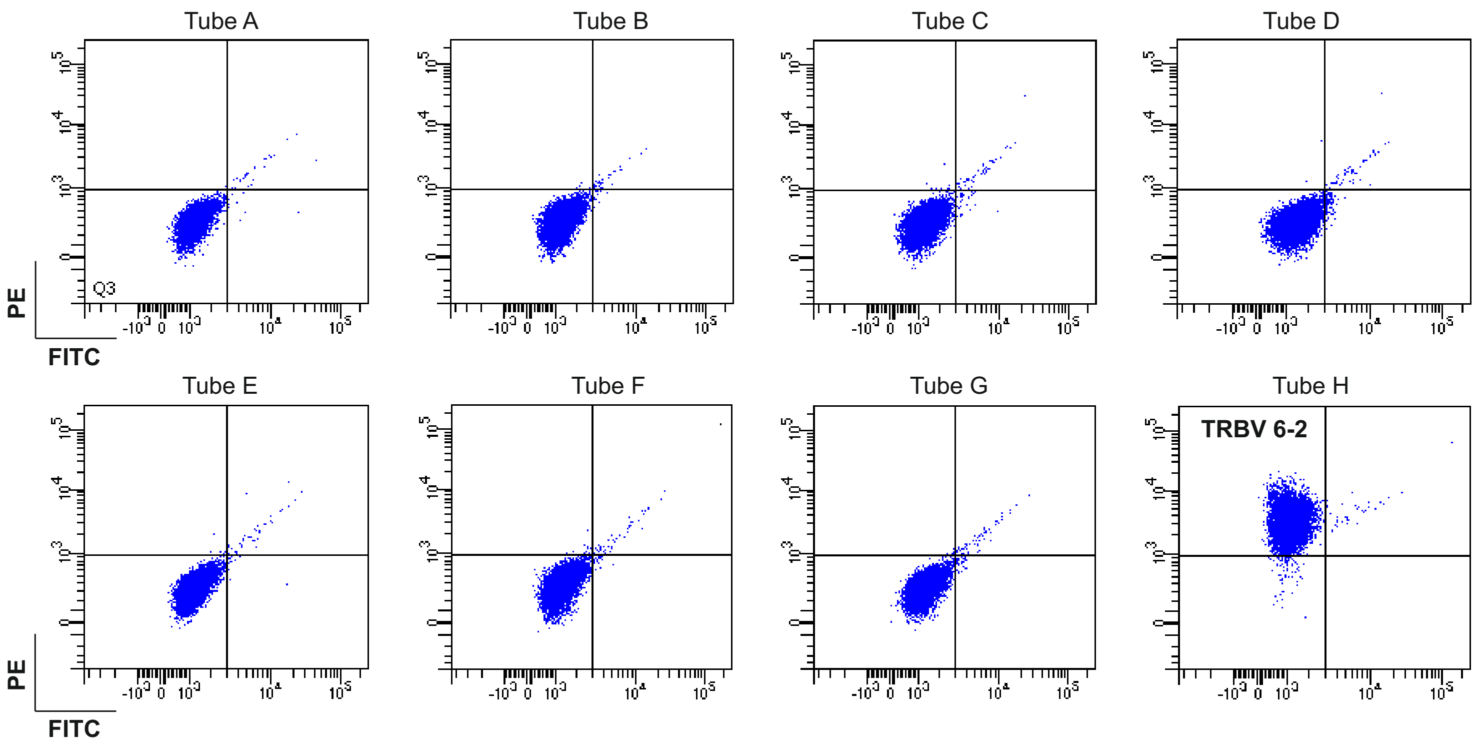

b

clone ID	Vα	Jα	CDR3seq Vα	CDR3seq N	CDR3seq Jα	Vβ	Dβ	Jβ	CDR3seq Vβ	CDR3seq N	CDR3seq Jβ
4,6,8,10	39	15	CAV	LL	NQAGTALIF	6-2	D2	2-7	CASS	GLAGRR	YEQYF
