# Supplementary material for: Immune monitoring and TCR sequencing of CD4 T cells in a long term responsive patient with metastasized pancreatic ductal carcinoma treated with individualized, neoepitope-derived multipeptide vaccines: a case report
Source: J Transl Med. 2018 Feb 6;16:23. doi: 10.1186/s12967-018-1382-1 (PMC5801813; doi:10.1186/s12967-018-1382-1)
Supplement: Supplementary file 5 — Additional file 5: Figure S5. Peptide-responsive clones at IM3 all displayed an CD45RA+ effector memory TEMRA phenotype. One representative example (clone #54) is shown in (a). In contrast, clones obtained from control cultures without peptide stimulation expressed a CD45RAneg effector memory phenotype, data by clone #6 are shown in (b). [file 12967_2018_1382_MOESM5_ESM.pdf]

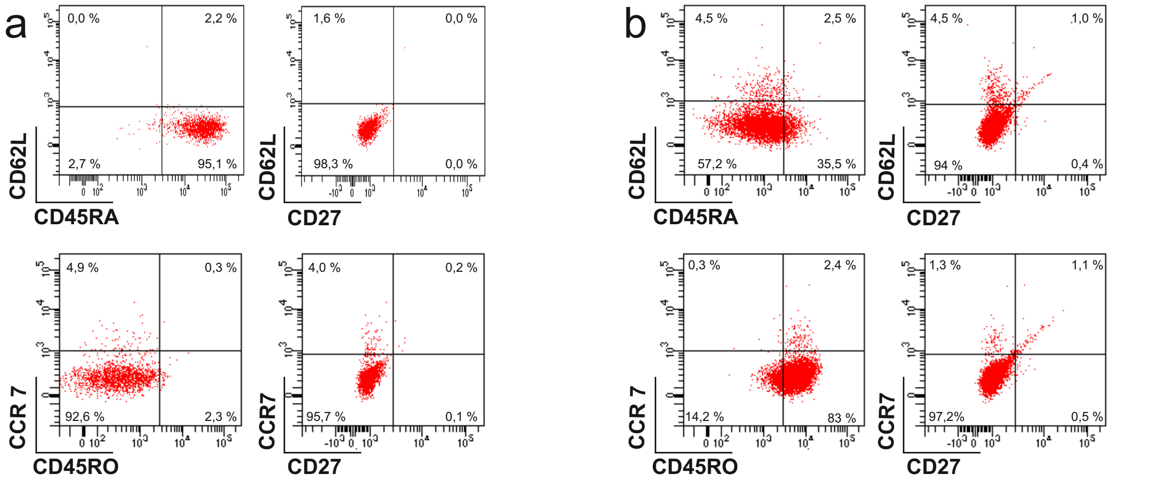

T_{em}: CD45RA$^+$, CD45RO$^-$, CD62L$^-$, CCR7$^-$, CD27$^-$

T_{eff}: CD45RA$^-$, CD45RO$^+$, CD62L$^-$, CCR7$^-$, CD27$^-$
